# Trehalose Activates Autophagy and Prevents Hydrogen Peroxide-Induced Apoptosis in the Bone Marrow Stromal Cells

**Published:** 2018

**Authors:** Shahram Darabi, Ali Noori-Zadeh, Hojjat Allah Abbaszadeh, Farzad Rajaei

**Affiliations:** a *Cellular and Molecular Research Center, Qazvin University of Medical Science, Qazvin, Iran. *; b *Department of Clinical Biochemistry, Faculty of Paramedicine, Ilam University of Medical Sciences, Ilam, Iran. *; c *Hearing Disorders Research Center, Loghman Hakim Medical Center, Shahid Beheshti University of Medical Sciences, Tehran, Iran. *; d *Department of Biology and Anatomical Sciences, School of Medicine, Shahid Beheshti University of Medical Sciences, Tehran, Iran.*

**Keywords:** Stress oxidative, Autophagy, Apoptosis, Bone marrow stromal cells, Trehalose

## Abstract

Bone marrow stromal stem cells (BMSCs) play a significant role in cell therapy. These cells quickly die after transplantation to the affected area due to oxidative stress. The natural disaccharide, trehalose which can be known as autophagy inducer. The present study aimed to investigate the role of trehalose in preventing BMSCs from oxidative stress caused by H_2_O_2_. BMSCs were isolated from the adult rats. The cells were divided into three groups: (a) control; (b) 100 µM H_2_O_2_; (c) 100 µM H_2_O_2_ and trehalose 3%. The morality rate was analyzed by viability test. Immunocytochemistry and Western blot was used in order to evaluate p62 protein and LC3II/LC3I ratio, respectively. In order to evaluate apoptosis, cleaved caspase-3 protein was used. In viability test, the survival rate for BMSCs after 8 h were 82%, 72%, 49%, and 39% (for groups who received 50, 100, 200, and 400 µM H_2_O_2_, respectively) compared to the control group. Pre-treatment with the use of trehalose 3% increased cell survivals. The levels of p62 protein, were increased in the cells under H_2_O_2_ treatment, while the levels of p62 protein in the cytoplasm, as autophagy inclusions, reduced for the group with trehalose pre-treatment. In addition, trehalose caused to increase LC3II/LC3I ratio and decreased the expression of cleaved caspase-3. Trehalose decreased apoptosis and increased the autophagy and survival levels of the cells against H_2_O_2_. Due to the unique properties of trehalose and its low toxicity, it can be used as a pharmaceutical agent in cellular transplantation to reduce oxidative stress.

## Introduction

BMSCs are able to differentiate into other ectodermal, mesodermal, and endodermal cell lines in laboratory culture medium (1-3). These cells, nowadays, play a significant role in cell and gene therapy (3), restorative medicine (4), and damaged tissue regeneration (5, 6). These cells are quickly dead after transplantation to the affected area due to hypoxia conditions, oxidative stress, and serum deprivation (7, 8). These cells, after transplantation to the injured spinal cord or the ischemic area of the brain, have very short lifespan due to oxidative factors and inflammatory cells (9-11). The ideas that prolong the cell life-span, by drugs therefore, play an important role in prolonging the survival of these cells after transplantation (12).

Trehalose is present in many microorganisms, plants, fungi, and insects (13). It is simply produced in the yeast, in response to environmental stressors including heat, cold, pressure, and lack of water (14). Trehalose is assumed to be a scaffold molecule that directly prevents protein aggregation (15). Trehalose was described as a chemical chaperone which stabilizes proteins, and prevents protein misfolding and protein aggregation (15). It was initially considered to be a rare sugar, but nowadays it is widely used in nature, pharmaceutical, and food industries. This sugar, due to its physiological and chemical properties, received a significant attention by many researchers (16). This sugar cannot simply pass through the cell membrane, while it can enter into cells through endocytosis and pinocytosis mechanisms, and has anti-inflammatory, anti-aggregation, and anti-aging properties (15, 17). Many studies have shown the role of trehalose in autophagic induction for neurodegenerative diseases (18).

Autophagy is a lysosomal pathway that degrades proteins and organelles, and recycles intracellular organelles and proteins to maintain energy homeostasis times of cellular stress (19, 20). In autophagy, the organelles and degraded fragments fall into double-membraned autophagosome. Autophagy is a process for maintaining cellular homeostasis under oxidative stress conditions. There is a relationship between deficiency in autophagy, and many neurodegenerative, cardiovascular diseases, aging and cancer (21). Some stressful states such as hypoxia and cell’s serum deprivation can induce autophagy and prolong cell life.

p62, a scaffold protein, has several domains whose function involve in the signal transferring process, proliferation, cell survival, death, tumor formation, and the response to oxidative stress (22). In the cell, ubiquitin–protein aggregation can cause neurodegenerative and cardiovascular disease. Lysosomal-autophagy process plays an essential role in removing cells from ubiquitin aggregation through the p62 ubiquitin-binding protein (22). p62, in addition to binding to ubiquitin protein, can be bounded to LC3 protein directing the ubiquitinated complex to the autophagosome to be degraded (23). LC3 is a substrate for initiating autophagy and forming autophagosome. In the studies conducted in this regard, the expression of LC3 was investigated as an indicator of autophagy (24). The conversion of LC3I to LC3II represents the autophagy process, and higher amount of LC3II shows increased formation of autophagic vacuoles in the cells (25).

The caspase-3 protein is a member of the cysteine–aspartic acid proteases family whose activation plays an important role in apoptosis (26-28). Caspases are formed as proenzymes and zymogens which are converted into large and small active subunits during proteolytic activity after activation (26).

In this study, we indicated that oxidative stress caused by H_2_O_2_ can reduce autophagy (reduced LC3II/LC3I ratio and increased p62) and increase apoptosis (expression of caspase-3 protein). However, the pre-treatment with the use of trehalose 3% can increase the autophagy and reduce apoptosis. Therefore, trehalose 3% can regulate the cellular defense system against cell death by activating autophagy. 

## Experimental

*BMSCs cultivation*

In the present study, four 6-8-week-old adult female Wistar rats from Pastor Institute were used. Observing ethical rules, rats were kept in a 12-hour light and dark under standard conditions of Qazvin University of medical sciences’ Animal House. After BMSCs isolation from the long bones of the lower limb, they were washed with sterile phosphate*-*buffered saline (PBS). The cells were cultured in Dulbecco›s modified Eagle›s medium: F12 (DMEM/F12: Gibco) containing, 100 U/mL penicillin/streptomycin (Gibco, BRL) and 10% heat-inactivated fetal bovine serum (FBS: Gibco, BRL) and incubated at 37 °C, 5% CO2, and 95% relative humidity (RH) until the third passage.

*Immunocytochemistry*

In order to investigate the mesenchymal origin of BMSCs at the third passage, 5000 cells were seeded equally into each well of the 24-well cell culture plate. The immunocytochemistry stages were performed according to the recommended ones with minor modifications (29). In summary, the cells were placed in paraformaldehyde solution 4% for 20 min and after being washed with phosphate buffered, the cells were placed in 0.3% Triton X for 15 min. After being washed with PBS, the cells were exposed to the primary antibody for 24 h at 4 °C. The primary antibodies include CD31 (endothelial stem cells marker), CD90 (mesenchymal stem cells marker), CD34 (hematopoietic stem cells marker), and p62 (autophagy marker) from ABCAM Company. The samples was washed with PBS and incubated with the secondary antibody conjugated with FITC (1:100; Chemicon) for 2 h at the room temperature. Nuclei were counterstained with PI (propidium iodide). Immunostaining was visualized under a fluorescent microscope (Olympus). The total number of the cells (PI staining) were estimated after counting at least three separate fields in the middle of each coverslip (200 cells per field counted).

*Viability test*

Prior to the study, viability test was performed by Trypan blue on cells in order to determine H_2_O_2_ toxicity and the protective effect of trehalose. The cells were divided into 1000 cells per well in the 96-well plate. In order to determine the H_2_O_2 _toxicity, survival rate for BMSCs was measured at different doses (0, 50, 100, 200 and 400 µM) for 8 h. After obtaining the lethal dose, the cells were divided into three groups: (A) control; (B) H_2_O_2_ and (C) H_2_O_2_ + trehalose. In order to examine the protective effects of trehalose, the cells were pretreated by trehalose 3% before being exposed to H_2_O_2_ medium (H_2_O_2_ + trehalose group). In order to analyze the survival rate of the cells, a volume of cell suspension and an equal volume of trypan blue were mixed and the cells were counted using the neobar lam under a microscope. In this method, the stain penetrates into the dead cells and turns the their color into blue. The non-stained cells represent the living cells. The percentage of the living cells can be obtained by counting the total number of the cells and the stained cells. Each cell group was counted three times using a microscope. 

*Western Blot*

The protective effect of trehalose against H_2_O_2_ damage was evaluated by analyzing apoptosis (Cleaved Caspase-3) and autophagy (LC3). After treatment of the cell groups as described, the protein of the cells was extracted and frozen at -80 °C for further use. The proteins were separated using SDS-PAGE gel (15%), and transferred to the polyvinylidene fluoride (PVDF) membrane. The PVDF membrane was blocked by fat-free dry milk (5%) for 30 min. Then, it was washed three times with TBST buffer and incubated in the solution containing peroxidase-conjugated secondary goat anti-mouse or -rabbit IgG (1:1000-1:2000) for 1 h. The membrane was washed three times using TBST buffer and the membrane image was developed using the enhanced chemiluminescence kit (ECL kit). Densitometric analysis was performed using Image J software. 

## Results

*BMSCs Analysis*

**Figure 1:**
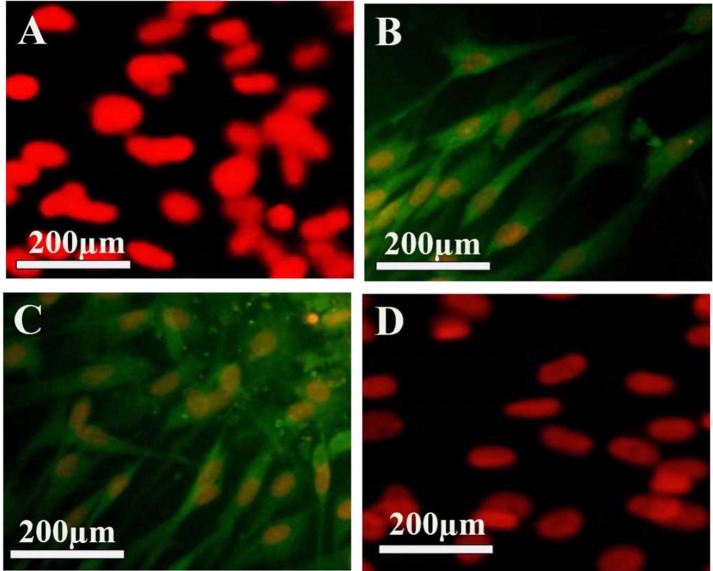
Characteristics of the isolated bone marrow stromal cells (BMSCs). (A–D) show BMSCs labeled with mouse monoclonal primary antibodies, incubated with anti-IgG mouse secondary antibody conjugated with FITC (secondary antibody), and counterstained with propidium iodide. (A) The primary antibodies used were anti-CD31, (B) anti-CD90, (C) antiCD106 and (D) anti-CD34.

BMSCs, after being isolated, had the appearance of round and spherical. They were stuck to the bottom of the flask after 48 h and after being washed with PBS, the floating cells that were not stuck on the bottom of the floor, were washed. After the third passage, BMSCs were evaluated by analyzing immunochemical surface markers in terms of mesenchymal origin. The cells were negative to CD31 and CD34, while indicating the positive immune response to CD90 and CD106 (Figure 1). The fluorescence light is associated with the conjugated secondary antibody to the FITC, which looks green. In order to count the number of the cells, PI dye was used, as it stains the nuclei (red color).

*BMSCs Viability*

**Figure 2:**
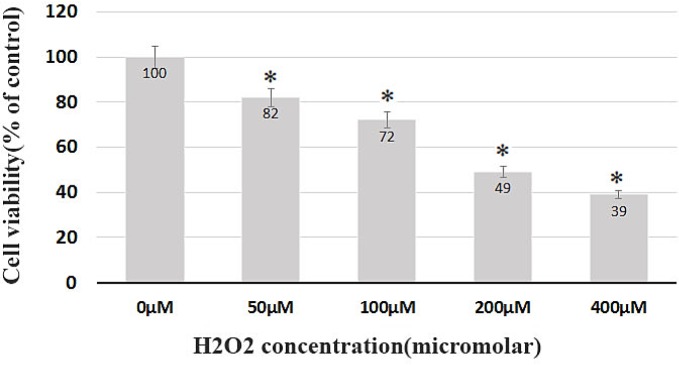
Dose-dependent cell death induced by H_2_O_2_ in BMSCs. Cell viability was determined by trypan blue assay. The survival rate after 8 h was 82%, 72%, 49% and 39% of the control group in the group receiving 50, 100, 200 and 400 μM of H_2_O_2_, respectively.

While testing the viability, the BMSCs at the third passage without the H_2_O_2_ treatment were considered as the control group. The cells were then treated with H_2_O_2_ at different concentrations for 8 h. The survival rate after 8 h was 82%, 72%, 49%, and 39% of the control group in the group receiving 50, 100, 200, and 400 μM of H_2_O_2_, respectively (Figure 2). The highest mortality rate belonged to the cell groups which received 200, 400 μM of H_2_O_2_, with a survival rate below 50%, and a significant difference (*p* < 0.05) with the control group. Such fatal doses were subsequently eliminated from the study (Figure 2).

**Figure 3:**
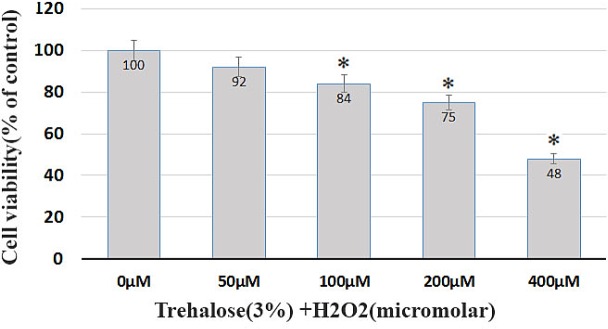
Cell viability. Trehalose pretreatment protects BMSCs against H_2_O_2_-induced cell death. The survival of the cells in the medium containing trehalose 3% and H_2_O_2_ at concentrations of 50, 100, 200, 400 μM was 92%, 84%, 75%, and 48%, respectively.

To evaluate the protective effect of trehalose, the BMSCs were pretreated with 3% trehalose 2 h before being exposed to H_2_O_2_ with the previous concentrations. The results showed that the pretreatment with trehalose increased cell survival. The survival of the cells in the medium containing trehalose 3% and H_2_O_2_ at concentrations of 50, 100, 200, and 400 μM was 92%, 84%, 75%, and 48%, respectively (Figure 3). The comparison of H_2_O_2_-exposed cells with and without trehalose 3%, as shown in Figures 2 and 3, reveals that trehalose significantly increases cell survival and decreases cell death compared to H_2_O_2_.

**Figure 4:**
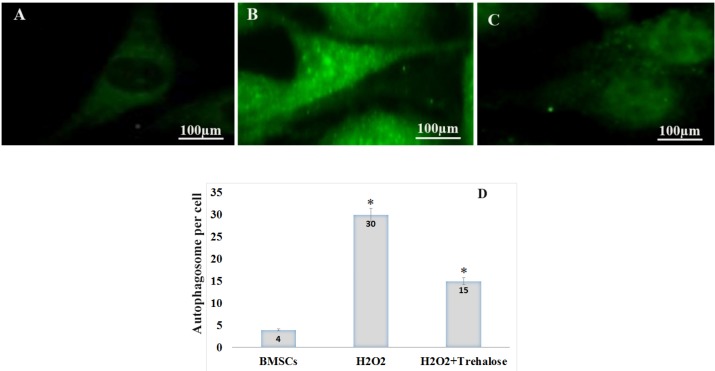
Effect of trehalose on autophagy in the cultured BMSCs. p62 immunostaining using an anti-p62 antibody showed the cytosolic accumulation of p62 in the BMSCs. (A) BMSCs, as the control group, were evaluated in terms of p62 protein expression at an environment free of H_2_O_2_. The expression of p62 protein was given in a basic and low form, with a diffuse distribution pattern throughout the cytoplasm. (B) BMSCs exposed to 100 μM of H_2_O_2_ for 8 h, the expression of p62 increased and p62 dot-like vesicles were formed in the cytoplasm. (C) BMSCs were pretreated with 3% trehalose, the number of glowing spots reduced compared to the H_2_O_2_ group. (D) After counting autophagic inclusions containing p62 in each cell, their number in the control groups, H_2_O_2_ and H_2_O_2_ + trekalose was 4%, 30% and 15%, respectively. BMSC were immunolabeled with anti-p62 primary antibody, incubated with FITC-conjugated secondary antibody.

**Figure 5:**
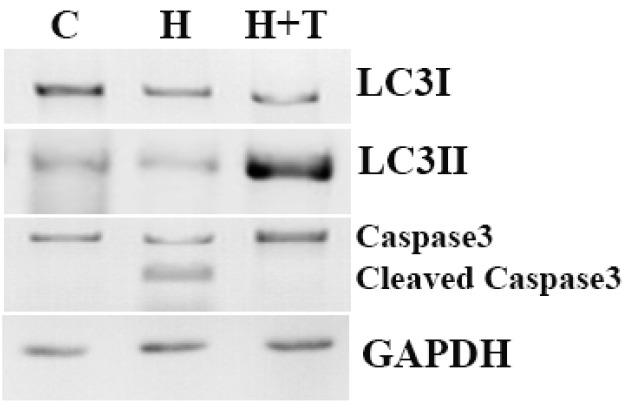
Western blot analyses of LC3, p62 and caspase-3 protein expression in control (lane1), H_2_O_2_ (lane2) and H_2_O_2_+trehalose (lane3) groups. Autophagic flux was examined by means of LC3 I/II conversion with the use of Western blot with LC3 antibody. LC3I, LC3II, p62 and Cleaved caspase 3 were detected using specific antibodies. GAPDH was used as an internal control. Caspase-3 was formed from a 32 kDa zymogen (inactive) that was cleaved into 17 kDa and 12 kDa subunits (active).

*H*_2_ *O*_2_ *caused autophagosomal vesicles formation containing p62*

p62 protein is an autophagy marker. p62 connects to LC3 and then decomposes through the lysosomal pathway of autophagy. Therefore, high levels of p62 represent a problem in autophagy. In autophagy, p62 is low. The study shows that p62 increases with raising the H_2_O_2 _concentrations such that the highest p62 in the H_2_O_2_ without trehalose treatment was shown after 8 h, indicating the inhibition of autophagy (Figure 4). Moreover, in order to evaluate the autophagy in BMSCs, the expression of p62 protein was scrutinized through immunocytochemistry in different groups (Figure 4). The formation of autophagosomes containing p62 characterizes the autophagic activity (30). First, the BMSCs, as the control group, were evaluated in terms of p62 expression at an environment free of H_2_O_2_. As shown in Figure 4A, in the control group, the expression of p62 protein was given in a basic and low form, with a diffuse distribution pattern throughout the cytoplasm. However, for the BMSCs exposed to 100 μM of H_2_O_2_ for 8 h, the expression of p62 increased and p62 vesicles were formed as glowing spots in the cytoplasm (Figure 4B). These points containing glowing vesicles indicate the formation of autophagosomes after the oxidative stress, and they outnumber the other groups according to the counting. In the H_2_O_2_ group pretreated with trehalose, the number of glowing spots reduced compared to the H_2_O_2_ group without pretreatment, indicating an increase in the autophagy (Figure 4C). After counting autophagic inclusions containing p62 in each cell, their number in the control groups, H_2_O2, and H_2_O_2_ + trehalose was 4%, 30%, and 15%, respectively (Figure 4D).

*The Role of trehalose in the autophagy induction and apoptosis prevention*

BMSCs, with and without trehalose, were exposed to H_2_O_2_ at 100 μM of concentration for 8 h to figure out if the oxidative stress caused by H_2_O_2_ activates the pathway of autophagy. The level of autophagy was assessed upon the determination of LC3II as the value of the LC3I. The ratio of LC3II to LC3I decreased in 8 h after being treated with 100 μM of H_2_O_2_, but in the medium of 3% trehalose and 100 μM of H_2_O_2_, this ratio increased (Figure 5). In order to examine the apoptosis in BMSCs, the proteolytic activity of cleaved caspase 3 was evaluated in different groups by means of Western Blot. Trehalose reduced the activity of the apoptotic marker of cleaved caspase 3 compared to H_2_O_2_. Figure 5 shows that trehalose decreased the activity of cleaved caspase 3 while its lack increased it and cleaved into tow subunit. Caspase-3, was formed from a 32 kDa zymogen (inactive) that was cleaved into 17 kDa and 12 kDa subunits (active forms) (Figure 5).

## Discussion

In summary, the study revealed that (a) 3% trehalose pre-treatment protects BMSCs against H_2_O_2_, and induces autophagy and decreases the death of the cells; (b) in the culture medium without trehalose, H_2_O_2_ causes apoptosis in the BMSCs in 8 h.

In normal physiological conditions, the cell retains the homeostasis through basic autophagy, using the removal of damaged or old organs, producing new organs and proteins (31). However, if autophagy cannot reactivate the basic hemostasis of the cell and adapt the cell to stress conditions, the path of apoptosis will be activated (32). The appropriate concentration of trehalose increases and regulates autophagy, but upon increasing trehalose concentration, the unwanted pathways of the cell which lead to its death are activated (33). Therefore, in this study, 3% trehalose was used because of the findings of the previous studies.

In this study, 3% trehalose increased the cellular survival.In this regard, previous studies have shown that the increase of the concentration of trehalose causes changes in osmolality of cells and their death (34). Other our finding was the rise in the autophagy activity because of trehalose. Trehalose is likely to prevent cells from death by increasing autophagy and eliminating the organs and proteins damaged by H_2_O_2_. In this study, H_2_O_2_ with a concentration more than 100 μM increased cell death. A variety of studies has shown the sensitivity of the cells to H_2_O_2_. For example, retinal pigment epithelia’s (RPE) are resistant to the concentrations more than 400 μM of H_2_O_2_ even until 24 h (35, 36), but neurons are highly susceptible to H_2_O_2_-induced oxidative stress and 10-50 μM of H_2_O_2_ leads to the toxicity of a neuron and so the autophagy, instead of playing a protective role, leads to cell death (37). In this study, in the trehalose-free environment and 100 μM of H_2_O_2_, the cells expressed the cleaved caspase 3 protein after 8 h, and the ratio of LC3II/LC3I, which is the autophagic index, decreased. The activation of caspases plays a major role in the cell death through apoptosis (38). In apoptosis, the executioner caspases (caspase-3/7 and caspase-6) begin with autophagy inhibitors (38). Many studies have shown the role of autophagy and apoptosis in stressful conditions and cellular signals leading to apoptosis in BMSCs. Caspase 3 is a protein from the family of cysteine-aspartic acid proteases, the activation of which plays an important role in apoptosis (38). The caspases (32 kDa) are pro-enzymes and zymogens which convert into two small (12 kDa) and large (17 kDa) active subunits through a proteolytic process after activation (39). After being broken, the enzyme activates caspases 6 and 7, and it, in turn, is activated by caspases 8, 9, and 10. This protein is the main caspase in beta-amyloid decomposition in Alzheimer›s disease (40). The caspases kill the cell through two outer (apoptotic ligands) and inner pathways (mitochondria). Caspase 3 is engaged in the formation of the brain by chromatin condensation and DNA fragmentation via apoptosis (41). The increased subunit of P17 in the bloodstream indicates myocardial infarction. Caspase 3 contributes to the differentiation of the stem cells of the blood and embryos (42). In previous studies, it has been shown that trehalose plays an important role in preventing apoptosis (43).

In many of the neurodegenerative diseases such as Huntington, 2% trehalose, after having been orally administered, traversed the blood barrier of the brain, and prevented the possible damage resulted from the poly-glutamine and poly-alanine in the cerebellum, leading to the improvement of movement and longevity (44). In fact, trehalose prevents the poly-glutamine-mediated protein aggregation. The ubiquitination of the p62 protein, which is an LC3-linked protein, generates protein compositions which could be removed with autophagy. Phosphatidyl ethanol amine, while being attached to L3, turns into LC3II, which is located in the internal and external membranes of autophagosomes, and it is completely eliminated after the attachment of autophagosomes to a lysosome. p62 plays a variety of cellular signals against stress, inflammation, and cell survival, and plays a role in the formation of ubiquitin-containing inclusions. Our study showed that trehalose reduced p62 levels in the cells.

Trehalose is a non-mTOR autophagy inducer that has been shown to reduce the toxic protein accumulation in the cells of many neurodegenerative diseases such as Alzheimer›s, Parkinson›s, Huntington›s through the induction of autophagy, and in the clinic, it has been shown to increase the life expectancy and relative improvement of patients. The special properties of trehalose and its low toxicity turned it into a pharmaceutical agent for the disorders requiring long-term medicine taking. In a study, it was revealed that trehalose protected *Candida albicans* against the oxidative stress caused by H_2_O_2_ (45).

## Conclusion

In the current study, the BMSCs subjected to cell death after exposing to H_2_O_2_, which was intensified after increasing the H_2_O_2_ concentrations. Furthermore, the protein expression in p62 in the cell cytoplasm increased, in accordance with other studies, indicating a decrease in autophagy level and an increase in the expression of antioxidant pathway genes. 
